# Use of Open-Source Large Language Models for Automatic Synthesis of the Entire Imaging Medical Records of Patients: A Feasibility Study

**DOI:** 10.3390/tomography11040047

**Published:** 2025-04-16

**Authors:** Fabio Mattiussi, Francesco Magoga, Simone Schiaffino, Vittorio Ferrari, Ermidio Rezzonico, Filippo Del Grande, Stefania Rizzo

**Affiliations:** 1 Imaging Institute of Southern Switzerland, Ente Ospedaliero Cantonale (EOC), 6900 Lugano, Switzerland; francesco.magoga@eoc.ch (F.M.); simone.schiaffino@eoc.ch (S.S.); ermidio.rezzonico@eoc.ch (E.R.); filippo.delgrande@eoc.ch (F.D.G.); stefania.rizzo@eoc.ch (S.R.); 2 Facoltà di Scienze Biomediche, Università della Svizzera italiana, 6900 Lugano, Switzerland; 3 Private Practice, 21100 Varese, Italy; vittferr55@gmail.com

**Keywords:** artificial intelligence, large language models, automatic synthesis of radiology reports, radiological workflow

## Abstract

Background/Objectives: Reviewing the entire history of imaging exams of a single patient’s records is an essential step in clinical practice, but it is time and resource consuming, with potential negative effects on workflow and on the quality of medical decisions. The main objective of this study was to evaluate the applicability of three open-source large language models (LLMs) for the automatic generation of concise summaries of patient’s imaging records. Secondary objectives were to assess correlations among the LLMs and to evaluate the length reduction provided by each model. Methods: Three state-of-the-art open-source large language models were selected: Llama 3.2 11B, Mistral 7B, and Falcon 7B. Each model was given a set of radiology reports. The summaries produced by the models were evaluated by two experienced radiologists and one experienced clinical physician using standardized metrics. Results: A variable number of radiological reports (n = 12–56) from four patients were selected and evaluated. The summaries generated by the three LLM showed a good level of accuracy compared with the information contained in the original reports, with positive ratings on both clinical relevance and ease of reference. According to the experts’ evaluations, the use of the summaries generated by LLMs could help to reduce the time spent on reviewing the previous imaging examinations performed, preserving the quality of clinical data. Conclusions: Our results suggest that LLMs are able to generate summaries of the imaging history of patients, and these summaries could improve radiology workflow making it easier to manage large volumes of reports.

## 1. Introduction

Radiology departments generate large amounts of clinical data on a daily basis, including radiological images, structured and unstructured textual reports, and clinical notes, that directly influence the diagnosis and treatment of patients in many different clinical settings [1,2]. On one hand, radiologists rarely interact directly with the patients whose radiological images they are reviewing due to time and resource constraints [3]. On the other, information about the patient’s medical history improves reporting performance and quality. Thus, radiologists need to read a high number of medical records during a reporting session. Furthermore, the increasing number of radiological examinations overtime [4] may even worsen the workload of radiologists when assessing the imaging evaluations of their patients.

In recent years, the development of large language models (LLMs) based on Transformer architectures, introduced initially by Vaswani et al. [5], has significantly advanced the state-of-the-art in natural language processing tasks. Models such as GPT (Generative Pre-trained Transformer) [6], BERT (Bidirectional Encoder Representations from Transformers) [7], and their derivatives have rapidly evolved [8], demonstrating impressive capabilities [9] in various complex linguistic tasks, including text summarization [10], translation [11], and question answering [12]. These advancements have opened new possibilities for applying LLMs in specialized domains such as medicine, where synthesizing and interpreting textual medical data accurately and efficiently is crucial. Within the medical field specifically, LLMs have been employed to facilitate tasks ranging from clinical documentation automation and report summarization, as demonstrated by the successful implementation of clinical summarization tools like RadLing [13] and AI-assisted decision-making systems such as MedPaLM [14], to decision support systems and personalized patient communication [14]. Such applications highlight the potential of LLMs to transform clinical workflows, enhance diagnostic accuracy, and ultimately improve patient care.

As a consequence, there is potential to advocate the preparation of a synthesis of previous medical reports using such advanced tools [15]. This would offer the possibility of saving valuable time while improving diagnostic accuracy when providing a consistent overview of a patient’s imaging findings. Although many state-of-the-art LLMs (e.g., GPT-4, Claude, and others) are proprietary, open-source models offer the advantage of customization and transparency, which are critical in healthcare settings where data privacy and regulatory compliance are paramount.

To the best of our knowledge, no previous study has evaluated the possibility of creating summaries of imaging studies using LLMs. Therefore, the main objective of this exploratory study was to evaluate the ability to generate automated summaries of radiology reports using three open-source LLMs—Llama 3.2 11B [16], Mistral 7B [17] and Falcon 7B [18]. Secondary objectives were to compare the correlations across the three models and to evaluate the length reduction provided by the three models.

## 2. Materials and Methods

**Scheme 1:**
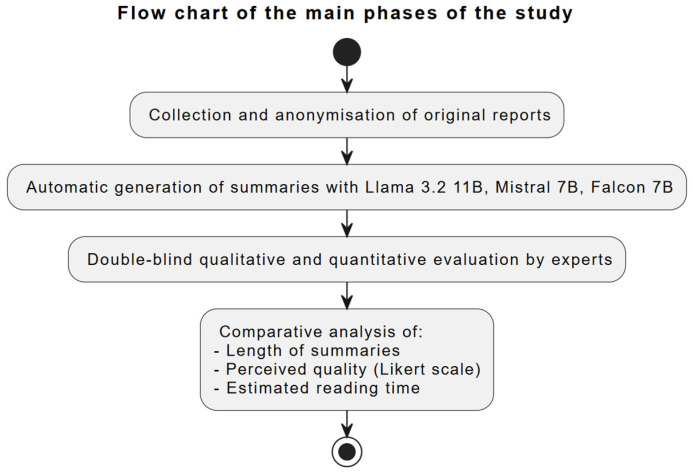
Flow chart schematically summarizing the main methodological steps of the study.

The study was conducted following a multi-stage process (Scheme 1). Initially, the original radiological reports of the patients were collected and anonymized. Subsequently, the reports were used as input to automatically generate summaries with three different large language models (LLMs). Subsequently, the produced summaries were subjected to double-blind qualitative and quantitative evaluation by clinical experts. Finally, the collected data were analyzed by comparing the length, perceived quality, and estimated reading time of the summaries with the original reports.

### 2.1. Data Collection and Filtering

All the radiology reports from four patients referred to the Imaging Institute of Southern Switzerland of the Ente Ospedaliero Cantonale (EOC) were included. Imaging examinations included conventional radiographs, computerized tomography, magnetic resonance imaging, ultrasound, positron emission tomography, other nuclear medicine examinations, neuroradiology, and radiological consultations. All data were anonymized following standard protocols, removing sensitive identifying information such as name, date of birth, and patient ID, and saved as text files.

The Ethics Committee of the Canton of Ticino (Switzerland) examined the study (Req-2024-01564) and decided that ethical approval was not required.

### 2.2. Models Used

To generate the report summaries, Llama 3.2 11B, Mistral 7B, and Falcon 7B models were used, all based on Transformer architectures and designed for natural language processing tasks. Each model was employed in its pre-trained version to create structured summaries of reports. Specifically, the models were downloaded and executed locally on dedicated computational resources using Python 3.12.3. The implementation leveraged the Hugging Face Transformers library along with PyTorch 2.6.0 as the backend, ensuring a controlled and reproducible environment. The experiments were carried out using Python 3.9 in a Docker container, and the processing was performed on a workstation equipped with two NVIDIA RTX 4060 GPUs (NVIDIA, Santa Clara, CA, USA). In addition, the input data underwent preliminary preprocessing steps such as tokenization and text normalization to ensure compatibility with the models.

### 2.3. Generation of Summaries and Prompt LLMs Used

Each model (Llama 3.2 11B, Mistral 7B, and Falcon 7B) processed all the patient’s reports, provided as textual input. For the generation of each summary, the three models were administered the same prompt, structured to require a clear, complete, and consistent summary of the reports.

Each of the three LLMs (Llama 3.2 11B, Mistral 7B, and Falcon 7B) was downloaded locally from Hugging Face repositories and run in a Python environment. Identical radiology reports were provided to each model in the same input format, along with a structured prompt (reproduced below). The models generated their summaries independently, with no information exchanged among them about the other outputs. This ensures that any differences in the generated summaries reflect the inherent characteristics of each model rather than variations in input or methodology.

The prompt was prepared as follows:

1. General Introduction

Begin with a brief paragraph summarizing the patient’s general health status. Specify whether the patient is completely healthy or if pathologies have been detected.

2. Detail by Anatomic District

For each anatomical district in which pathology was detected, create a separate section with the following format: Anatomical District Name.

- Pathologies Detected: List the pathologies identified in this district and provide a brief description of each pathology.
- Examinations Performed: List all examinations related to this anatomical district in chronological order from oldest to most recent. Give dates of examinations in DD/MM/YYYYY format if available.
- Trend Summary: Describe the evolution of the pathologies over time, based on the reports. Highlight any significant changes.

3. Absence of Pathologies

Only if no pathologies are found in the reports, write clearly that nothing abnormal was found; otherwise, skip this point.

4. Format and Presentation

Use titles in grass for sections and anatomical districts. Organize content clearly and consistently to facilitate reading. Do not use asterisks, bullet points, or unnecessary symbols.

5. Tone and Language

Maintain a professional and objective tone. Use clear and concise language, avoiding terms that are too technical unless essential.

### 2.4. Double-Blind Experts Evaluation

After the three summaries were generated, they underwent a blinded assessment by three doctors, along with the original files.

Experts evaluation: Three physicians—two radiologists with 13 and 19 years of experience and one clinical physician with 39 years of experience—blindly evaluated the anonymized files of the patients. Each radiologist evaluated the three summaries for one patient, while the clinician evaluated the files of two patients. To ensure evaluator blinding, each summary was anonymously labelled with letters (A, B, C), each corresponding to a specific LLM, and provided to the evaluators without revealing the actual identity of the model. In this way, none of the evaluators were aware of which LLM had generated the summary they were examining. The patients were randomly assigned to the evaluators, and the original reports for each patient were presented chronologically, respecting the clinical history. Evaluators first read all original reports and subsequently reviewed all three summaries before providing their evaluation.

After reviewing the imaging reports as single text files of each imaging examination, the experts evaluated the summaries generated by the LLMs and filled in a quantitative rating for each of the following four criteria:

1. Completeness of information (any omissions from the original reports);
2. Clinical accuracy (presence of correct information or errors in interpretation);
3. Narrative coherence (text structure, logical connection between different examinations);
4. Compliance with radiological terminology (use of correct and appropriate technical terms).

The evaluations were performed according to a Likert Scale [19] where 1 = very reliable and 5 = not reliable.

Finally, each expert provided a qualitative assessment highlighting the strengths and weaknesses of the summaries for each model-generated summary.

### 2.5. Length of Summaries and Reading Time

To assess the time efficiency of summaries compared to full reports, the text reduction percentage was calculated using the following formula:

(1) $$Reduction\;percentage=(1-\frac{Words\;in\;the\;summary}{Words\;in\;the\;full\;report})\times 100$$

The reduction percentage in word count achieved by the summary compared to the full original report was calculated. The percentage is computed by subtracting the ratio of the number of words in the summary to the number of words in the full report from 1, then multiplying by 100.

The results were analyzed to determine the relationship between the length of the summary and the perceived quality of the evaluators. Quality was assessed using a 5-point scale, considering parameters such as completeness, clinical accuracy, and narrative coherence. In addition, correlation coefficients were calculated between the length of the summary and the quality scores assigned by the reviewers.

To assess the time saved in reading the summaries compared to the full reports, we used the results described in the study [20], where reading duration was calculated based on the total number of words, using an average speed of 210.5 words per minute. Next, the length between the reading of the full reports and the summaries produced by the three models was compared.

### 2.6. Comparison of LLM Performances

In order to statistically compare the performance of the three LLMs, namely, Llama 3.2 11B, Mistral 7B, and Falcon 7B, an in-depth statistical analysis was performed on the physicians’ ratings using the scores assigned according to the Likert scale.

The difference between the mean scores of the models was evaluated using the nonparametric Kruskal–Wallis statistical test.

The Kruskal–Wallis statistical formula used is as follows:

$$H=(N-1)\frac{\sum_{i=1}^{g}n_{i}{\left(r_{i}-r\right)}^{2}\;}{\sum_{i=1}^{g}\sum_{j=1}^{n_{i}}{\left(r_{ij}-r\right)}^{2}}$$

where

*N* is the total number of observations across all groups;
*g* is the number of groups;
*n_i_* is the number of observations in group *i*;
*r_i_* is the average rank of all observations in group *i;*
*r_ij_* is the rank (among all observations) of observation *j* from group *i;*
*r* is the average of all the *r_ij_*.

## 3. Results

The comparative analysis of the evaluations provided by the three doctors showed significant differences between the summaries produced by the three models, as described below.

### 3.1. Quantitative Evaluation of the Summaries Generated by the LLMs

**Figure 1:**
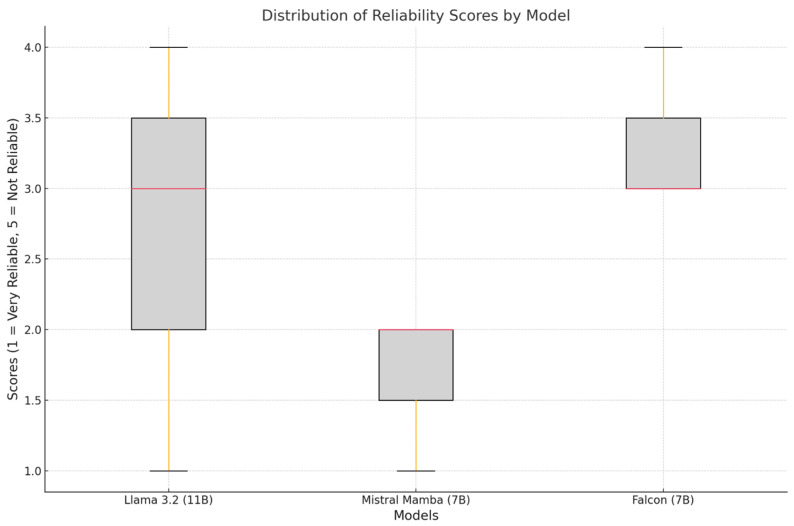
Distribution of the reliability scores of the three models.

Figure 1 represents the distribution of reliability scores (1 = very reliable, 5 = unreliable) for each model. The error bars indicate the standard deviation. Mistral (7B) was the most reliable model (mean: 1.67 ± 0.58). Falcon (7B) received the highest score, indicating lower reliability (mean: 3.33 ± 0.58). Llama 3.2 (11B) showed a higher variability (mean: 2.67 ± 1.53).

**Figure 2:**
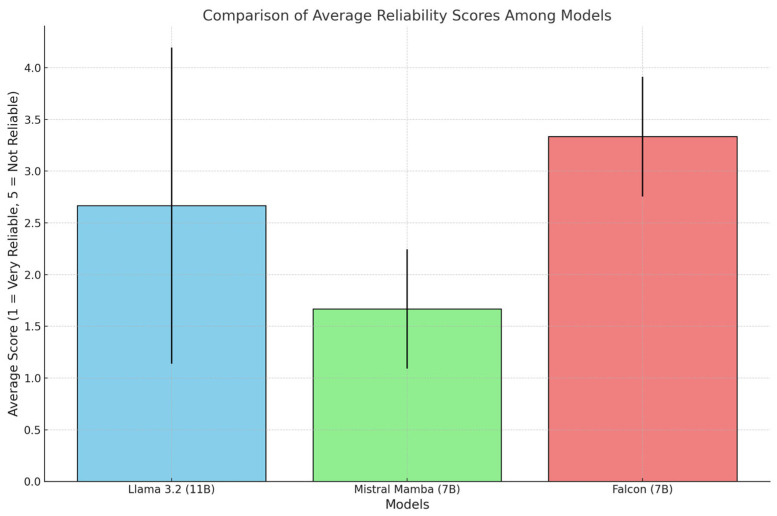
Average reliability scores with standard deviation.

Figure 2 shows the distribution of scores assigned to the models, highlighting median, range, and outliers.

Mistral (7B) showed a narrow and consistent distribution of scores, reflecting uniform ratings by physicians. Llama 3.2 (11B) showed a wider distribution of scores, indicating a lack of consensus among evaluating physicians. Falcon (7B) showed a distribution of scores suggesting occasional outliers, reflecting inconsistencies in its summaries.

### 3.2. Qualitative Evaluation of the Summaries Generated by the LLMs

Llama 3.2 11B generally provided detailed and informative summaries, showed a very good adherence to radiological terminology, and included many correct technical details. However, in some cases, the narrative structure exhibited minor repetitions or inconsistencies. This accounts for the greater variability in the scores attributed, compared to the other models.

Mistral 7B stood out for its consistency and fluency of exposition, produced fluent and well-organized summaries, and was particularly appreciated for its readability. Therefore, this model showed the most consistent distribution of scores and the lowest average reliability scores (Figure 1 and Figure 2).

Falcon 7B provided concise summaries in line with the main information in the reports, with good lexical accuracy, although sometimes not perfectly aligned with standard radiological terminology. This model, while maintaining acceptable accuracy, ranked as the least reliable model with the highest average reliability score (Figure 2).

### 3.3. Model Correlations

**Figure 3:**
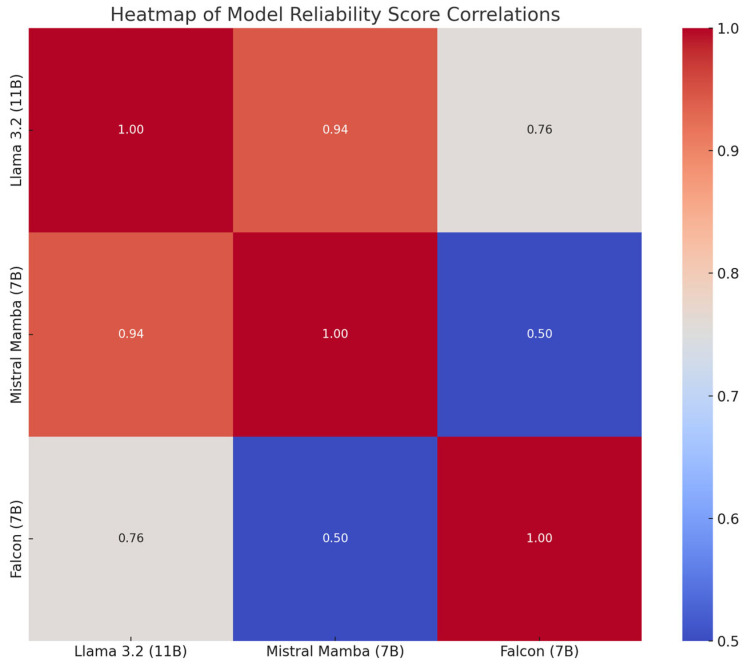
Heatmap of models correlations.

The graph in Figure 3 shows the correlations between the reliability scores assigned to the models, where higher values indicate greater similarity in the ratings.

Mistral (7B) correlated moderately with Llama 3.2 (11B), suggesting overlapping strengths in reliability. Falcon (7B) showed weaker correlations with the other models, reflecting different performance profiles. Llama 3.2 (11B) showed partial overlap with both models, but maintained a distinct performance profile.

**Figure 4:**
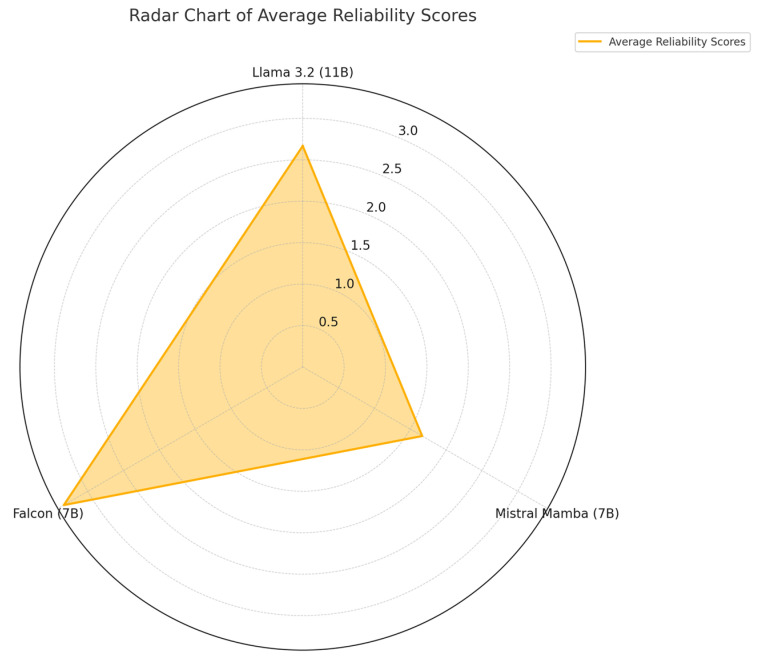
Radar chart for visual comparison of averages.

Figure 4 presents the average reliability scores (1 = very reliable, 5 = unreliable) on a common scale, highlighting the relative differences among the models.

Mistral (7B) outperformed the others with the lowest average score in all categories. Falcon (7B) showed higher scores than the other two models. Llama 3.2 (11B) maintained a balanced position, but its ratings showed high variability.

Overall, the results confirmed the ability of LLMs models to generate accurate and consistent radiological summaries without any specific additional fine-tuning. The use of a double-blind evaluation protocol reduced the risk of bias and allowed a more reliable comparison of the performance of the different LLMs.

### 3.4. Length of Summaries and Reading Time

The length of the summaries generated by the three models showed high differences, as shown in Table 1.

**Figure 5:**
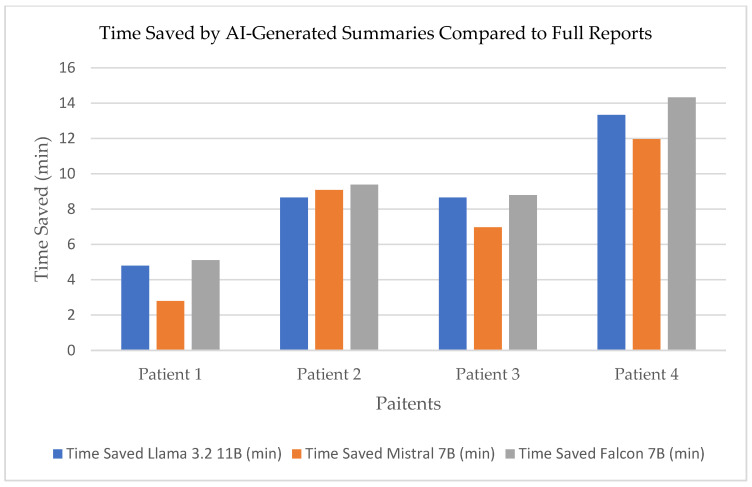
Time saved in minutes when reading summaries compared to full reports.

Time saved in reading the summaries generated by the three models compared to the full reports is shown Figure 5.

The percentage of word reduction compared to the original reports is shown in Table 2.

Falcon 7B provided the greatest compression of content, providing concise summaries with a good level of accuracy, whereas Mistral 7B produced longer but more detailed summaries, resulting in a smoother but less concise reading. Llama 3.2 11B showed a length reduction that maintained a balance between length and completeness.

Correlation analysis between summary length and quality scores revealed that more concise summaries tended to be less detailed, while longer summaries were considered redundant by evaluating physicians.

### 3.5. Comparison of LLM Performances

Applying the Kruskal–Wallis statistical test to the data collected in the study yielded the following values: sum of ranks Llama 3.2 11B: 16.5; sum of ranks Mistral 7B: 6.0; sum of ranks Falcon 7B: 22.5.

The H-test result was compared with the critical value of the X^2^ distribution with two degrees of freedom and was ~6.2 (*p* < 0.05).

Therefore, the results show that the Mistral 7B model represents the most reliable solution among the three models tested.

## 4. Discussion

The adoption of open-source LLMs to summarize imaging reports represents a significant opportunity to improve clinical workflow efficiency [21]. In light of the average reading speeds reported by Brysbaert [20], which provide a benchmark for physicians’ text processing speed, the reduction in text volume achieved through automated summarization could translate into significant time savings during report review. For example, models that produce more concise summaries, such as the Falcon 7B, achieve a substantial reduction in word count, and this could allow physicians to process information faster, potentially reducing the overall time to read the patient’s entire report history. Previous studies [22] have demonstrated that summarizing clinical reports significantly improves reading speed and reduces cognitive fatigue among radiologists.

In parallel, our analysis revealed a trade-off between brevity and the preservation of complete clinical details. While highly condensed summaries improve efficiency, models that generate longer, more detailed texts may better preserve diagnostic information. Finding the optimal balance between brevity and completeness is essential to ensure that summaries remain user-friendly and clinically reliable. Indeed, the potential operational benefits of reducing text length must be balanced against the need to maintain high standards of accuracy and clinical safety. Concerns regarding information omission in automated summaries have been similarly highlighted in the literature [23], emphasizing the importance of ensuring accurate clinical details are preserved. Future studies could specifically evaluate clinician preferences for summary length, potentially using controlled trials to systematically assess the optimal balance between brevity and completeness. Incorporating real-time clinical feedback and continuous updates to the model could help align automated results with current medical practices and terminologies.

The accuracy and consistency of the summaries generated are crucial; in some cases, key clinical details may be omitted or presented with variable terminology. As noted by Ghosh et al. [24] in the development of RadLing, the performance of LLMs is closely related to the quality and scope of the training data. Furthermore, the integration of these synthesis tools into existing RIS/PACS systems remains an area that requires further contextual evaluation within individual healthcare institutions. The integration of multimodal clinical data, including electronic health records (EHR), has shown potential to enhance diagnostic precision and personalization in AI-driven clinical reports [24].

Furthermore, it would be ideal to create models capable of automatically generating reports not only on the basis of imaging results, but also by integrating patient-specific parameters such as age, medical history, laboratory results, and previous medical interventions. Such integrated models could improve the clinical relevance and customization of automatically generated summaries, providing more contextually accurate and clinically useful reports. This is a promising direction for future research.

This exploratory study has some limitations. First, the number of evaluators and patients included was a limitation. We acknowledge that this is an important limitation, and that before applying a LLM to create a summary of the imaging reports of a patient, larger studies should be reported. To address this, future research should measure actual reading and task completion times in a realistic clinical workflow, possibly employing direct observational or time-tracking methodologies. However, this was a feasibility study to assess if the LLMs are able to perform such a task, and we demonstrated that it is indeed possible. Therefore, from now on, we do expect that more studies will evaluate the performance of LLMs in this kind of task, even beyond the imaging summaries, such as summaries of the metabolic medical history or the oncological history. Second, in this study, we assume that the marked reduction in length of summaries would allow faster evaluation of the imaging history of the patient, but we performed this analysis according to an estimated time, rather than an effective time. However, the time spent in reading text reports is clearly affected by the number of words and by the length of the entire text. Another limitation is that we did not estimate the additional overhead in time reduction related to skipping the handling of multiple documents, compared to opening a single summary created by an LLM. We expect that such an analysis would have reinforced the advantage of using a single LLM summary over multiple documents. However, it would have required a larger sample of patients and accurate measurement of operating time, possibly tracked using a stopwatch controlled by another person. Since this was an exploratory study, we did not plan for this measurement, but we will include it in future, larger studies. Furthermore, the readers were provided the reports and summaries to evaluate as files outside the usual tools used in the radiological clinical practice, such as the radiology information system (RIS) and the picture archiving and communication system (PACS). Future implementations might integrate the summarization tools directly into existing RIS/PACS infrastructure, allowing assessment of real-world usability and clinical utility as well as seamless incorporation into clinician workflows. Previous literature has identified integration challenges and stressed the importance of workflow compatibility as essential for the effective implementation of AI tools within RIS/PACS systems [21]. Lastly, another limitation concerns the language of the reports used. In our hospital, radiology reports are mainly written in Italian. Consequently, this feasibility study was carried out exclusively on reports written in Italian. Therefore, future studies should explore and validate the performance of the models on texts written in different languages, thereby assessing their generalizability.

This may be important for the actual usage of these models, possibly including also the possibility of real-time feedback mechanisms from clinicians, in order to iteratively refine the quality and clinical relevance of the summarized information. Indeed, even a highly performing model, if not integrated in the hospital information systems, may be unhelpful and reverse itself to a time-consuming task.

## 5. Conclusions

In conclusion, the integration of large language models (LLMs) for the synthesis of imaging reports represents significant progress toward increasing clinical workflow efficiency and improving patient care. This exploratory study demonstrated that automated synthesis of imaging reports using open-source LLM can substantially reduce the length of reports, potentially leading to significant time savings in clinical practice. However, it also identified a critical trade-off between brevity and preservation of essential clinical details, highlighting the need to carefully balance these factors to maintain diagnostic accuracy. Furthermore, the integration of patient-specific clinical data, such as medical history and laboratory results, promises to increase the clinical relevance and accuracy of automated summaries. The study also acknowledged limitations related to language and system integration, emphasizing the importance of adapting these tools to various language contexts and ensuring seamless integration into existing hospital information systems (RIS/PACS).

## Figures and Tables

**Table 1:** Number of reports and word count of reports and summaries.

Patient	Number of Reports	Total Words in Reports	Syntheses Llama 3.2 11B	Syntheses Mistral 7B	Syntheses Falcon 7B
1	14	1314	306	727	240
2	12	2164	343	254	190
3	44	2094	274	629	243
4	56	3182	377	665	167

**Table 2:** Word count reduction percentage.

Patient	Llama 3.2 11B Reduction (%)	Mistral 7B Reduction (%)	Falcon 7B Reduction (%)
1	76.70	44.67	81.73
2	84.14	88.26	91.22
3	86.91	69.95	88.39
4	88.15	79.10	94.75

## Data Availability

The dataset used in this study was derived from clinical data owned by the authors and has been fully anonymized. However, the data are not publicly available due to patient confidentiality and ethical restrictions. The three large language models used (Mistral, Llama, and Falcon) can be accessed at https://mistral.ai/ (accessed on 12 November 2024), https://www.llama.com/ (accessed on 12 November 2024), and https://falconllm.tii.ae/ (accessed on 12 November 2024), respectively. There are no plans for future data releases. For further inquiries, please contact the corresponding author.
